# Selections and Abstracts

**Published:** 1901-12

**Authors:** 


### SELECTIONS AND ABSTRACTS.

### The Etiology and Symptomatology of Constipation, Including the Differential Diagnosis Between Atonic and Spastic Constipation.*

Constipation may be best defined as that condition in which there is an imperfect emptying of the bowels. It may result from many different diseases. Though only a symptom of some pathologic state, either in the innervation or muscular apparatus of the intestines, or of disease elsewhere in the body, it is exceedingly prevalent in civilized communities, and in its chronic form, is almost never cured by the administration of medicines alone nor by any directly opening measures, whether in the form of laxative drugs, per os or rectum, or colon douches, or even the usual routine massage.

*Etiology.*—To enumerate all the diseases which seem often to stand in a casual relation to constipation would almost exhaust the list of important known maladies. Prominent among those which nearly always produce constipation (and some of them complete obstruction) are meningitis, brain tumors and many other cerebral and spinal affections, lead poisoning, volvulus, invagination of the intestines, hernia, peritonitis, appendicitis, abdominal and pelvic tumors, etc. It is also a frequent result of blood impoverishment and of most depressing diseases of the nervous system, inflammation of the stomach or upper intestines, ulcer or tumor of the same, and stricture of the bowels; also, of abnormalities in the gastric secretion, especially hyperchlorhydria, many diseases of the liver and pancreas, and particularly of the downward displacement (ptoses) of the abdominal viscera, as well as backward displacement of the uterus and other diseases of the pelvic organs. Prolapse of the right kidney (movable kidney), which is exceedingly common in women, is often a factor in the production of constipa-

*Read by Boardman Reed, M.D., before the Philadelphia County Medical Society, September 11, 1901.

tion by obstructing at times the lumen of the duodenum, and the agglutination of folds of intestine to each other or to neighboring structures may seriously impede the onward propulsion of the feces.

The most prolific causes of chronic habitual constipation, and those most amenable to non-surgical treatment, are to be found in either one of two opposite conditions involving both the nervous and the muscular apparatus of the gastro-intestinal tract, and requiring quite different methods of treatment. These are atony and spasm. Atony of the stomach walls, whether amounting to dilatation or only to motor insufficiency with delayed empting, results generally in a deranged intestinal peristalsis showing itself usually at first in the form of constipation. Atony in any part of the intestine must manifestly produce a like result.

A cramp or spastic state of the pylorus or of the muscles of the intestines, leading to irregular local tonic contractions of the circular fibres, is a common and often unrecognized cause of constipation. In hysteria and in certain forms of neurasthenia such localized spasms are perhaps almost as frequently responsible for difficult defecation as muscular atony, and much more frequently than any other single cause. It is probable also that the deranged digestion, both gastric and intestinal, which so often accompanies neurasthenia by producing fermentation and abnormally acid conditions in the alimentary canal, conduces powerfully to the spasmodic action; and it is likely that portions of the bowel, the mucous membrane of which is in a state of chronic catarrhal inflammation, have an increased tendency to spastic contractions.

Authorities differ widely as to the relative importance of various factors in the causation of both atonic and spastic constipation. Glènard considered displacements of the stomach and intestines as chiefly responsible, while Emminghaus traces habitual constipation to degenerative changes in the splanchnics, and Dunin thinks it attributable mainly to central functional anomalies in the nervous system. Boas* finds it difficult either to deny or confirm these theories, but points out that in any fully developed case of neurasthenia with constipation there may be found a vicious circle,

---

*Diagnostik u. Therapie der Darmkrankheiten. Leipzig, 1899.

and he aptly illustrates his idea by describing as follows a supposed case, such as we all often see :

"A previously healthy woman begins to suffer with constipation and requires aperients. Gradually these become inefficient; defecation is more and more difficult and imperfect. At the same time, there is taken a decreased amount of nourishment, either in consequence of a misuse of purgatives or as a therapeutic measure ('easily digestible food'), or as a result of a bad general condition, or from anæmia or gastric derangements, *e.g.*, atony. Naturally, then, there follow emaciation, dropping of the abdominal viscera and, with these, increase of the constipation, and finally, as a capstone to all these symptoms, the picture of well-marked neurasthenia. Here, as every experienced physician must concede, the enteroptosis is not the cause, but the consequence, of the habitual constipation, and the same holds good also for the neurasthenia. But, on the other hand, the loss of flesh from whatever cause can lead to the development of visceral displacements and so produce constipation, or, perhaps more correctly, favor its development, as also, in like manner, genuine neurasthenia (according to Dunin's views) may prove the basis for the development often of even very stubborn forms of constipation."

An insufficient amount of food or drink, a too bland diet lacking in refuse matters, or a too predominately nitrogenous diet, sedentary occupations, deficient exercise, and a want of regularity in going to stool are further important causes of chronic atonic constipation.

*Symptomatology.* It has been denied that auto-intoxication can be caused by dry feces, no matter how long retained, and in Germany the resulting phenomena are more generally considered to be reflex; but however accounted for, some of the following symptoms may be constantly observed in constipation : Dizziness, headache, insomnia, mental hebetude or depression, nausea, furred tongue, offensive breath, flatulency, colics, failing appetite, as well as other indications of impaired digestion, various affections of the skin, including particularly urticaria, and often, objectively ascertained, deranged gastric secretion (especially excessive HCl) and probably lowered gastric motility, as well as indicanuria and excess of the aromatic sulphates, and of the total acids in the urine. Other objective signs are dry, hard stools, lumpy or made up of agglutinated balls of different color and consistency, or hard globular feces of various sizes, from that of hazel-nuts up, or in spastic cases, as well as in cases of organic stricture, feces molded in narrow cylindric forms.

Periodic transient attacks of diarrhea, with usually mingled lumps and eiquid feces, which are often exceedingly offensive, may be considered as a symptom of chronic constipation. In these cases there is irritation of both the mucous membrane and musculature of the intestines by the long continued pressure of the hard fecal masses and distention from the imprisoned gases, and probably a catarrhal process is also set up in places, through infection from the enormously multiplied bacteria in the stagnant feces, with abundant formation of organic acids from fermentation.

It is a serious mistake to treat such diarrheal attacks by opiates and astringents, or even by antiseptics alone, when nature has so clearly pointed the way to a prompt clearing out of the intestines which should be accomplished preferably by a colon douche. Recurrent diarrheas, with either no stools or insufficient stools between, are signs of chronic atonic constipation, or else of a mild chronic enteritis, and need to be treated accordingly.

*Diagnosis.* It is of chief practical importance to differentiate the atonic and spastic forms of constipation from each other, and both of these from the organic changes which may impede defecation. Westphalen,* of St. Petersburg, has written elaborate papers concerning both the atonic and spastic form of constipation and has pointed out the differences between the two very clearly. Numerous authors, including Nothnagel, Boas, Flick, Fleiner and others, in Germany especially, have mentioned the spastic as a possible form of constipation, but with a few exceptions do not lay much stress upon it. Many cases of this affection, which were seen in my own earlier practice, were not recognized as such. They were often given abdominal massage, not only without benefit but with the result of increasing their constipation. These were patients suffering from neurasthenia in connection with gas-

***Archiv f. Verdauungskrankheiten*, vol. vi., No. 2, and vol, vii. Nos. 1 and 2.

tro-intestinal derangements, and many of them showed spastically contracted abdominal muscles and excessive knee jerks. I do not now prescribe the usual vigorous abdominal massage for such patients, though I sometimes find them to be benefitted by light stroking—effleurage ; but try to cure them by general roborant measures, trusting to diet, special exercises, oil enemas, etc., to keep their bowels open. Besides the fact that spastic constipation occurs in nervous, excitable patients with heightened reflexes, Westphalen points out that in such cases the stools, though usually complete, are passed with the greatest difficulty and often only after much straining and long delay. Afterward there is left behind an unsatisfactory feeling, as though the rectum had not been perfectly emptied, even when an examination would show that no feces remained there. This sensation he considers to be due to an irritated condition of the nerve-endings in the rectum. A like hyperesthesia of the mucous membrane of the bowel is supposed to play a part in producing the irregular contractions of the circular muscles which hinder the onward progress of the feces. A contrary group of symptoms obtains in atonic constipation. Here the amount of feces passed daily is too small, not at all in proportion to the quantity of food taken, and yet the patient may pass them with little straining or effort, and feel afterward as though he had had a thorough evacuation. He may also be neurasthenic, but will not likely have such exaggerated reflexes. His abdominal muscles will not be so rigid upon palpation, and a finger introduced into the rectum will not be so tightly grasped.

With regard to the appearance of the stools in the two forms, all agree that small ones of lead-pencil or little-finger shape and size mean a spastic contraction of some portion of the bowel, when an organic stricture can be excluded, except in cases of semi-starvation, especially in cancer, as a result of which the lumen of the intestine may become greatly lessened. These stools may be canaliculated. I may add as an important diagnostic point observed by myself, that spastic conditions are almost never constant, but the patients will at some time (often as a result of nerve sedatives), pass stools of normal calibre, whereas, when there is a permanent stricture, the stools are always in either a slender form or fluid.

Westphalen also lays some stress upon the consistency of the stools, insisting that while they are very hard and dry and often covered with a thin layer of mucus in the atonic form, those passed in the spastic form contain no mucus outside or inside, but have usually a larger percentage of water and are tougher, more sticky and of a more glistening appearance—*glänzend.* The latter also contain less gas, so that they will not float in water as normal feces or those from a case of atonic constipation usually will.

Careful palpation and percussion over the abdomen will show differences between the atonic and spastic types of constipation. In the former a more general tympany should be demonstrable, with possibly masses of hard feces to be felt in the cecum or flexures of the colon; while in the latter type one may often feel portions of the intestine contracted like a cord under the finger. Then, in the spastic form, too, there are more frequently very sensitive spots in various parts of the abdomen, especially in the region of the umbilicus, and there are portions over which marked tympany contrasts sharply with the duller note of adjoining regions, showing imprisoned gas. Tabetic patients in an early stage are likely to present the phenomena of spastic constipation. Judging from my observation, it is highly probable that most neurasthenic patients who suffer excessively from intestinal flatulence which accumulates in places to their great discomfort, finally passing with borborygmi through evidently narrowed coils of intestine, while the feces themselves are retained, have the spastic form of constipation, however complicated with other conditions. Finally you may find both forms in the same case, since it is quite possible for atonic constipation to be complicated at times by a spastic condition.

From the constipation or obstruction dependent upon serious organic disease, such as strictures, tumors, inflammatory affections, etc., both the atonic and spastic form of impeded defecation can generally be differentiated by thorough and painstaking examinations, with the help of our modern exact methods. The above mentioned hint as to the constant smallness of the stools in organic stricture should be helpful. Filling the stomach with gas and again with liquid and afterward, if necessary, treating the colon in the same way, will often reveal latent tumors and their attachment, and a thorough douching of the colon with warm water will usually demonstrate a chronic enteritis by the amount of mucus brought away.

The importance of taking the necessary pains to reach a definite diagnosis in these cases cannot be overestimated. The time has gone by when we should be content to let our constipated patients continue indefinitely with whatever laxative drug affords them the most comfortable evacuations. Our duty is to ascertain the exact cause of the constipation, and then to cure it, as is possible in a large majority of cases.

---

### TREATMENT OF THE EYE BY THE GENERAL PRACTITIONER.*

There is probably no organ of the body that the general practitioner is better qualified to treat than the eye. There is hardly a disease or condition to which flesh is heir but what he is ready to give advice and treatment. But when the visual organ is affected, he at once loses confidence in himself, and very frequently tells his patient that he knows nothing about the eye. I think that this is a wrong condition for the medical man to be in. It is an injustice to your patients not to be able to correctly diagnose the common diseases of the eye and to properly treat many of them. Ophthalmology is not a separate science, but simply one branch of medical knowledge. You certainly should be able to early recognize the dangerous diseases, and if you did not feel qualified to treat them, the patient could be quickly sent to an oculist.

There are in the United States, according to the census of 1890, over 50,000 persons who are totally blind, and, I am sorry to say, Vermont has the highest ratio of any State in the Union. It is generally conceded that forty per cent. of all cases of blindness could have been prevented by proper treatment. You will, therefore, see the great responsibility which rests upon you to properly care for these patients in the first stages, as you are, in the great majority of cases, the first to see them.

We are becoming more and more impressed every year with the

---

*Read by George H. Gorham, M.D., Bellows Falls, Vt., at the Vermont State Medical Society.

importance of preventive medicine. This will apply in a large degree to diseases of the eye. You have all heard undoubtedly of the Credé method of treating the eye of the new-born child with a strong solution of nitrate of silver, but I presume that only a very few present have practiced it.

At the time that Credé originated this method ten per cent. of all cases in his lying-in hospital were afflicted with ophthalmia neonatorum; at present there are only 0.02 per cent. I do not think that it is necessary in most instances to follow this treatment, but I see cases of purulent ophthalmia often enough to think that the attending physician should use the silver solution in all cases that are in the least suspicious. A one-per-cent. solution is generally strong enough. Washing the eye with a 1 : 5000 bichloride solution is also good, or even a saline solution. Remember that over one-fourth of all cases of blindness in the civilized world are due to this disease. Quite a number of the States and countries have passed laws fining any midwife, or attendant, who neglects to properly report any suspicious cases of red or inflamed eyes in the new-born infant. Vermont has neglected to do so.

I would next call your attention to the hygiene of the eye, especially in reference to the school life of children. I would advise the examination of the eyes of all children at the time that they first entered upon their school work. This could be done by the superintendent. All children whose distant vision did not reach a certain standard and whose near point was abnormal, or who had inflamed eyes or lids, should be sent home with a note to their parents advising them to consult their family physician, or an oculist, at once. In Munich they make three or four examinations a year at regular intervals with beneficial results, the standard of vision constantly rising. In the past but very little attention has been paid to the location of our schoolhouses or the amount of light per room, but in the modern structures this has been rectified.

It is during this period of life, the school age, that we see so many cases of asthenopia, or eye-strain. I consider the treatment of this condition one of the most important that is brought to your attention, for not only the immediate happiness and well-being of the patient is involved, but future generations are liable to be affected.

Every person at birth is hypermetropic, or far-sighted, but under the constant strain of civilized life a large number become astigmatic, and later myopic or near-sighted. A myopic eye, you must remember, is a diseased eye, and your endeavor should be to so guard and protect your young patients that they will not advance to this condition. This can be brought about by first looking after their hygienic surroundings, and secondarily by correcting all errors of refraction or eye-strain. By the present mode of instruction a child is under a constant strain with his eyes from the time he enters the first grade until he completes his education.

Is it any wonder that many suffer from asthenopia? These cases I should advise sending to a competent oculist, for a large majority can be relieved by properly fitted lenses. The busy physician has not the time to devote to it, even if he had had the training to do it. By so doing, at the first symptoms manifested—pains through the eyes, headaches, and weakness of the eyes on study—you will not only be able in a large number of cases to give your patient relief from his symptoms, but will also prevent an advancement of his refractive error. Statistics compiled by both Risley and Oliver prove that if these cases are treated early it will prevent their development to a higher or more advanced condition. It is a positive fact that there are fewer cases of myopia to-day relatively than a generation ago, and the reason is that the children have had their low degrees of refraction properly treated.

The physicians of to-day are well aware that many of the functional disturbances of the nervous system are due to a strain on the ciliary muscle of the eye, or an unbalanced condition of the extrinsic muscles of the eyeball. I do not believe that even the proper correction of all abnormal conditions in the eye will cure every case of neurasthenia, epilepsy, or hysteria, but I do know that in very many of these cases you will give relief, and help in others. It is your duty to use and apply all forms of treatment that will be of help to your patient, and I certainly believe that the correction of all errors in refraction and placing the external muscles of the eye in equilibrium will go far toward it.

One of the most frequent conditions of the eye which you are called upon to treat is a foreign body upon the conjunctiva or cornea. I would urge upon you the importance of making a careful examination in such cases, as I have frequently found small particles upon the cornea, generally of a vegetable nature, which had remained there for some weeks. In a few cases sympathetic inflammation had already set in. In many of these cases a small tuft of cotton upon an applicator will easily remove the object; four-per-cent. solution of cocaine to be used unless the patient has symptoms of glaucoma. I think that general practitioners, as a rule, use too strong solutions of the astringents for the different kinds and degrees of conjunctivitis. A one-grain-to-the-ounce solution of zinc sulphate will be more efficacious in many cases than one or two or three grains. The same will apply to silver solutions.

In acute conjunctivitis I very frequently use a tannic glycerin solution, about four grains of the former and one drachm of the latter to the ounce of aqua destillata.

Do not diagnose all chronic or subacute inflammations of the conjunctiva as granulated lids. It is a comparatively rare disease here in the country. The bluestone still has its advocates for this condition. I have used the strong tannic acid solution, nitrate of silver, and subconjunctival injections of bichloride of mercury 1:1000. Whatever else you prescribe do not use poultices of any form except in some lid inflammations; they will simply congest the conjunctiva and make the inflammation worse. Hot boric acid solutions are nearly always permissible except in injuries and acute purulent ophthalmia, when cold applications and icebags are better.

In all inflammations of the cornea, especially any form of ulceration, use hot applications frequently repeated; at the same time prescribe atropine, two grains to the ounce. If you have a central corneal ulcer I should advise you to have your patient consult some oculist, as in many of these cases there is an opacity which is liable to permanently remain and obstruct vision. It is well to have some one else to share the responsibility with you.

I presume that in acute inflammations of the eye more mistakes have been made in diagnosis between conjunctivitis, iritis, and glaucoma than in all the other diseases of this organ combined. In some cases it is difficult to distinguish between them. You must remember that in conjunctivitis the superficial vessels only are dilated and injected, the pain is not severe, the cornea, pupil, and iris are in a normal condition, and the vision good except from occasional deposits of secretion. In iritis, on the other hand, the deeper blood-vessels are engorged with blood, the pain is generally sharp and severe, the pupil contracted, the iris of a muddy brown or greenish color, aqueous humor slightly turbid, vision somewhat impaired. Tension of the eye is normal in both diseases. In glaucoma the conjunctiva is deeply injected, of a dusky-red color; veins dilated; pain very severe through eyes and temples, anterior chamber shallow; partial anesthesia of the corena, which has a cloudy appearance; pupil dilated, very marked; diminution of vision, and a pronounced increased tension of the eyeball.

Having these diagnostic points in mind it is not often that you will make a mistake in your diagnosis.

Hot applications and atropine are your sheet-anchor in iritis. Two grains to the ounce is usually sufficient, but do not be afraid to use four or even eight grains to the ounce for a short period, using constitutional treatment according to cause of the disease.

In cases of glaucoma I think that it is always advisable to consult an oculist. In most cases an iridectomy is demanded, although eserine is of temporary relief in some instances.

I think that every well equipped physician should become familiar enough with the use of the opthalmoscope so that he could at least discover any gross changes which had taken place in the posterior eye. By so doing he would be able not only to treat the constitutional disorders which are producing the eye symptoms, but in many cases he would send the patient to a specialist before injury had been done. Frequently we find an albuminuric retinitis when there have been no marked symptoms of kidney disease. Almost the first positive proof of tumor in the brain is seen in the choked disk of the optic nerve. The same will apply to atrophy of the optic nerve, indicating a serious spinal trouble, or toxic condition of the nervous system. If you have a patient who uses tobacco or spirituous liquors to excess and complains to you in regard to his vision, you should at once suspect atrophy of the nerve. He should have treatment as early as possible in order to preserve his vision, or at least part of it. As there are more cases of blindness due to nerve atrophy than any other cause, you should be on your guard against hastily treating a case of marked impairment of vision without first giving your patient an ophthalmoscopic examination, or having it done.

In regard to operations on the eye, I think that only exceptionally, and those of a minor nature, or in case of emergency, should these be attempted by a man doing general practice. He is not called upon frequently enough to have that fine technique which is required in many cases.

As I have before stated, errors of refraction and an unequal strength of the external muscles of the eye are the cause of many functional nervous troubles and reflex conditions. And, on the other hand, many constitutional diseases produce local ocular disturbances. In a strumous condition of the system we get styes, blepharitis, and phlyctenulæ; in rheumatism, iritis and choroiditis; in syphilis and tabes, paralysis of the external muscles of the eye and nerve atrophy.

Syphilis is the cause of three-fourths of all cases of atrophy and choroiditis, one-half of iritis, and one-third of ocular paresis. In chronic Bright's disease we have a retinitis, and in diphtheria a paralysis of the muscles.

I think that every practicing physician should have a set of Snellen's test types in his office, and be able to learn the visual acuity of his patients who complain of imperfect vision. I want to impress upon you that this test does not reveal to you whether he needs lenses to correct any error of refraction, or prisms to relieve strain. In many cases of asthenopia the patient has vision above normal. It will simply give you an idea as to his sight.

A subject which extends over as large a field as this cannot be covered by a paper limited in time. But I urge upon you the importance of making a more careful research and study of this subject. It will be a source of gratification to you and your patients, and I trust to future generations.—*Medical Age.*

### THE SCIENTIFIC STUDY OF ACCIDENTS.*

The experiences of accident companies in dealing with the various businesses and occupations of the world have been reduced to a fairly scientific basis. It is now possible for these companies to foretell approximately the number and variety of accidents that men in certain businesses are exposed to, and fix their rate of insurance in such a way as to give a reasonable return upon the capital invested. The later idea of insurance of health is now in process of experimentation, but this phase of insurance has not been settled by any means, and it is possible that it will never be as satisfactorily arranged as the question of accidents, for an accident is a certain definite occurrence, like death itself, while sickness is a varying quality which admits of much malingering, if not actual deception.

It is interesting to look over the manuals prepared for insurrance agents by their companies. These books rarely get into the hands of the public, or even of the medical profession, and, therefore, the information that they contain is largely unknown to the medical profession.

It must be remembered that the classifications of occupations are not made up according to fancy or personal predilection, but are the result of the data of a long record of accidents.

In the first place, taking the prohibited list of businesses which are not insurable at all against accidents, we find naturally that manufacturers, users, or custodians of nitroglycerine, dynamite, or its compounds, cannot be insuréd against accidents; neither can powder-mill employés, cartridge or percussion-cap makers, or pyrotechnists. This is only natural, and the public, if called upon to name non-insurable businesses, would, undoubtedly, place these first of all upon the list. But there are other businesses which the accident companies regard as unfavorable, as making nitroglycerine or gunpowder. In the first place, soldiers in barracks or field service are regarded as equally ineligible, while the professional baseball player is viewed with as much disdain as the handler of dynamite. Submarine divers, æronauts, circus riders and United

* By J. Howe Adams, M.D., Philadelphia, Pa., in the *Medical Fortnightly*.

States deputies supervising illicit distilleries, are all considered to be taking their lives in their hands in the pursuance of their daily work.

The next class of men whose work is considered so dangerous that they require special contracts in which they are limited to $250 in case of death, for which they must pay the comparatively enormous premium of $30 annually, are brakemen on freight trains or in yards, couplers, miners, and trimmers of electric light carbons.

After this special class comes the provision known technically as "special hazardous," in which the insured must pay $25 a year and be limited to $1,000 in case of death. Cowboys, cattle tenders, buzz-sawyers, hunters, lumbermen, sawmill employés, and employés of furniture, sash, blind and door manufacturers running machinery.

The next technical division is that of "extra-hazardous," who are compelled to pay $20, and are limited generally to $1,500 for insurance in the average company. In this division come the majority of railroad employes, such as conductors on all kinds of trains, locomotive firemen and engineers, bridge builders and bridge carpenters. It also includes all sailors and such special businesses, as well as diggers, wood choppers, windmill builders and employés in iron and steel manufactures. Thus it will be seen that a sailor runs no more risk of accident than a train conductor.

The next class technically considered is the "hazardous" occupation, who must pay at the rate of $15 for $1,000, and are generally limited to $1,500 in case of death. This division contains many surprises, for the farmer and farm laborer is regarded as doing work as liable to accident as the fireman in the paid city departments. The common laborer comes in this class, as does the dairyman, the coal heaver, the ice man, the telegraphic or telephonic lineman, the quarryman, the cornice setter. There are also a number of the well-known trades which come under this division; these are the blacksmith, the butcher, the carpenter, the professional fisherman and sea pilot. The fact that the captain and mate on all classes of vessels have a better chance for life and limb is recognized by putting them in this class instead of the "extra hazardous." For some reason or other, on account of his ability, the train baggage-master is put in this class, recognizing him as a better risk than the conductor or the engineer.

We now come to a class of businesses which drop the ominous word "hazardous" and are called "extra medium." This does not avail them much, for they are still limited to $1,500, for which they must pay $12.50 annually. This includes bartenders, cable and electric road employés, celluloid workers, distillery employés, volunteer city firemen, employes in glass factories and hostlers.

We now begin to come down to the ordinary risks. The next class rejoices in the title of "medium;" they are limited to $2,000 insurance in case of death, for which they must pay at the rate of $10 a thousand annually. This includes a great many of the ordinary businesses, such as house-painters, boat-builders, book-binders, bottlers, cabinet-makers, chocolate-makers, all kinds of ferrymen, makers of agricultural implements, makers of bicycles, attendants on the insane. It also includes such railroad employés as work around the track or station, such as car-painters, car-cleaners, air-brake inspectors, truckmen at the station, etc. It also includes the men who travel habitually on Pullman trains, such as the cook in dining or hotel cars, porters and Pullman conductors. It is interesting to note that these trainmen are less exposed to accident than ordinary trainmen.

The next division of risks is that of "extraordinary," in which the amount is limited to $2,000, to cost at the rate of $8.50 a thousand. This division is rather an exclusive one, and contains such occupations as the superintendent of furniture manufacturers, who, while not actually running the machinery, directs and makes repairs.

The next type would probably be not over-pleasant to hear themselves set down in insurance terms as "ordinary" risks. It includes such businesses as the bartender in a first-class hotel, bill-posters, bookbinder, boiler-inspector, superintendent of building, candle-maker, carver, gilder, civil engineer doing railroad work, private coachman, custom-house inspector handling trunks, dyer, elevator man, etcher, fireman for stationary engine, gardener, glacier, goldbeater, harness-maker, hydraulic engineer, huckster, working jeweler, lithographer, mail service employé on railroads, paper-hanger, professional cook, peddler, plasterer, porter, tailor, watchman, and wood-carver.

Now comes the class of risks known as "extra-preferred," who need only to pay $6 a thousand, and are permitted generally to insure up to the amount of $5,000 in one company. This class of men are those who are generally superintendents, supervising contractors, supervising proprietors or manufacturers in the various lines of business. Insurance companies regard the men who supervise the work of their employés personally as not as good risks as if they confine themselves simply to office work.

The next two classes which are considered the cream of the accident business, are the "preferred" and the "select," who need pay only $5 and $4 respectively a thousand, the difference being that the select is allowed twice the maximum amount of the preferred for death as a rule. This includes men doing office work as a rule, such as architects, artists, lawyers, auctioneers, authors, barbers, bookkeepers, stock-brokers, all kind of clerks, commission merchants, druggists, editors, grain-dealers, hotel-keepers, insurance agents, all kinds of proprietors or manufacturers who do office work only, all publishers, and such railway men as auditors, treasurers, superintendents, and the higher grade of officials who do not travel regularly; also ministers, typewriters.

I have purposely omitted all reference to physicians, leaving that to the last. Accident companies regard the physician as a better risk than the surgeon, and they regard the city physician better than his brother practitioner in the country. As the city physician is regarded as the best risk, he is put in the best class, the select; next comes the physician and surgeon with city practice only; he is placed in the preferred class, while the country physician and surgeon is placed down in the classification as "ordinary," which is the fourth from the top, for which he must pay twice as much as his city brother, and be limited to half the amount of indemnity in case of accident or death by accident. This is the cold-blooded view which the accident companies take of the dangers of the country doctor's life.

### IS GONORRHEA CURABLE?

This is a question that has been very much discussed by the medical profession of late years. The finding of the germ of gonorrhea in so many uterine appendages that have been removed from women whose husbands are apparently free from any infection has led to the query whether a man, who has once had a gonorrhea, is ever entirely rid of the diplococcus.

At the last meeting of the American Medical Association a committee was appointed to obtain the opinions of the leading specialists of this country and Europe regarding the prognosis and treatment of gonorrhea in the male. Neisser, of Breslau, replied that he thought the disease curable in all properly treated cases, that cases of anterior specific urethritis were as a rule easily amenable to treatment, but that posterior urethritis, on account of its relative inaccessibility and the frequency of prostatic involvement as its accompaniment, was much more serious. Complete recovery could only be obtained through prolonged treatment and the giving of careful attention to the condition of the prostate gland.

Andry, of Toulouse, gives a favorable opinion, thinking all cases curable where the patient is willing to care for himself, as required, and as long a time as is necessary. Andry relies principally upon the lavage method of Janet.

Tommasoli, of Palermo, is of the opinion that gonorrhea is absolutely curable, so far that the patient can be authorized to marry.

The answers received from American specialists were of a like character, all agreeing that a gonorrhea, if proper treatment has been followed, is curable.

The question which the general practictioner has so often to face is, when is the disease cured? Patients will show absolutely no more of the usual symptoms, and still there may be lurking in folds of the urethra a few gonococci which may lie dormant for an indefinite period. Probably the only safe method of determining this question is the urine. If, after repeated examinations, the urine shows an absence of shreds, glandular or prostatic, or if the shreds are of so slight a character that they dissolve in a short time, then may the individual be pronounced thoroughly cured. This method ought always to be followed before advising a patient that his cure has been effected, but physicians will continue to encounter failure in the treatment of gonorrhea until such time as the laity have been educated up to the gravity of this affection. At present it is not regarded by them as a very serious affection. Progress can only be made by impressing upon each and every individual so affected what his future may be, and of the woes that may befall the woman he marries.—*Medical Age.*

---

### "MEMORIA IN AETERNA."

As time flies by, amid the rush and bustle of this eminently practical, work-a-day world, one unconsciously displaces from the mind of today the remembrances of the happenings of yesterday ; there are, however, some things which should remain "in everlasting remembrance." The gentleness, strength and beauty of the personal character of William McKinley and the inestimable value of his services to the nation and the world at large, should not be consigned to the mental dust heap of oblivion, but should be cherished as a precious heritage by every patriotic American, whether native or foreign-born. Feeling confident that their friends in the medical profession will appreciate at its proper worth a souvenir which shall serve as a constant reminder of the life, character and services of our third martyr President, The Arlington Chemical Company has prepared for gratuitous distribution a magnificent enlarged reproduction (17x13) of one of the finest and most faithful portraits in existence. Competent critics who have seen this reproduction have expressed themselves as surprised at the faithfulness with which the beautiful Rembrandt effect has been carried out with its rich, dark sepia tints and with the general artistic worthiness of the portrait as a whole. The advertisement of Liquid Peptonoids is so unobtrusive as to be entirely unobjectionable. The Arlington Chemical Co., Yonkers, N. Y., will be pleased to send a copy to any physician who may have failed to receive one, together with suggestions for proper method of framing.

